# How to Wash the Baby

**Published:** 1900-11

**Authors:** Lydia Pinkham Maginnis


### How To Wash the Baby.

As a rule babies should be washed by hand. Machine-washed babies do not mature so rapidly, and being rubbed on a washboard is likely to wear holes in the baby.

The baby should be put to soak over night in lukewarm water in which has been dissolved a handful of pearline. This takes the place of the old-fashioned way of leaving him in a wash-boiler on the stove for three or four hours and saves much labor.

In the morning, after rinsing in three waters, add a little ball blue to the last and pass the baby quickly through it. Too much bluing is apt to give the baby a faded look.

We do not think that babies should be put through the wringer too soon after washing. Better results are obtained by hanging them on the line at once, if it be a sunny day. Otherwise they may be hung up before the kitchen fire, and when thoroughly dry starched and ironed at your convenience.—*Mrs. Lydia Pinkham Maginnis, in "Life."*

---

*This paper is published herewith.—Ed.

